# Photodynamic Diagnosis for the Identification of Intestinal-Type Gastric Cancers and High-Grade Adenomas

**DOI:** 10.3389/fonc.2022.861868

**Published:** 2022-05-02

**Authors:** Hiroki Kurumi, Takuki Sakaguchi, Keiichi Hashiguchi, Taro Yamashita, Masashi Fujii, Yuichiro Ikebuchi, Akira Yoshida, Hajime Isomoto

**Affiliations:** ^1^ Division of Gastroenterology and Nephrology, Faculty of Medicine, Tottori University, Yonago, Japan; ^2^ Department of Endoscopy, Nagasaki University Hospital, Nagasaki, Japan

**Keywords:** gastric cancer, gastric adenoma, photodynamic diagnosis, 5-aminolevulinic acid, endoscopy

## Abstract

Gastric cancer is the second most common cancer in Japan. The incidence of gastric cancer remains high owing to the increase in the elderly population. Endoscopy outperforms radiography in identifying early gastric cancer (EGC). Furthermore, image-enhanced endoscopy (IEE) has been developed and implemented worldwide in clinical practice. Magnifying IEE images can help to visualize the microvascular pattern and microstructure architecture, which is used for the characterization of EGC. However, accurate endoscopic diagnosis requires the experience and skill of endoscopists, making an objective and simple diagnostic method desirable. In this retrospective study, we investigated the diagnostic yield of 5-aminolevulinic acid (5-ALA)-mediated photodynamic diagnosis (PDD) for identifying gastric cancers and high-grade adenomas. In total, 52 lesions from 43 patients were ultimately included in the study. We detected 5-ALA-mediated protoporphyrin IX fluorescence in 45 of the 52 lesions that were initially intended for PDD, resulting in a detection rate of 86.5%, whereas each signet ring cell carcinoma was negative using 5-ALA PDD. In eight of the patients with multiple lesions, 17 lesions were identified using 5-ALA PDD. Again, we took biopsies from six areas that we suspected as new lesions. While 4 lesions were gastric neoplasms resected by endoscopic submucosal dissection, two other lesions were normal. Preoperative 5-ALA-PDD could provide additional diagnostic yields to detect such multiple lesions simultaneously. No severe adverse events were observed. Prospective multicenter studies are warranted to confirm the usefulness of 5-ALA PDD for EGC identification.

## Introduction

Gastric cancer is the fifth most common cancer and fourth leading cause of cancer-related deaths worldwide (1). *Helicobacter pylori* (*H. pylori*) infection is considered the main cause of gastric cancer (2, 3). Despite a reduction in the number of *H. pylori* infections, the number of gastric cancer cases continues to increase owing to an increase in the elderly population, and the number of deaths has plateaued. Currently, the number of gastric cancer cases ranks second among all cancers in Japan with the number of deaths caused by gastric cancer ranking third (4). The incidence and mortality rates of gastric cancer make it a critical public health problem.

Endoscopy outperforms radiography in identifying early gastric cancer (EGC), which can be curatively treated *via* endoscopic resection in cases of nominal risk of lymph nodal metastasis. Image-enhanced endoscopy (IEE) has been developed and implemented worldwide in clinical practice. In particular, magnifying endoscopic images can be helpful to visualize the microvascular pattern and microstructure architecture for the characterization of EGC. However, an accurate endoscopic diagnosis requires the experience and skill of endoscopists (5, 6). Thus, there are still limitations associated with standardizing diagnostic capacity using magnifying IEE that can be shared among novice to board-certified expert endoscopists. Thus, a more objective and simple diagnostic method is warranted.

Photodynamic diagnosis (PDD) is used to detect tumors *via* illumination with a specific wavelength of light to produce fluorescence after the administration of specific photosensitizers that accumulate in tumors *in situ*. In recent years, PDD using 5-aminolevulinic acid (5-ALA) has been reported for some cancers, including gastric cancer, bladder cancer, and brain tumors (7, 8). Regarding the metabolic mechanism underlying the processing of 5-ALA, it is taken up intracellularly into the cytoplasm *via* oligopeptide transporter 1, which is a peptide transporter, and then transported to the mitochondria and excreted extracellularly by ATP-binding cassette transporter G2. In cancer cells, the heterocyclic organic compound protoporphyrin IX (PpIX) markedly accumulates as a result of abnormal transporter activity in the cell membrane and mitochondria, as well as enzyme abnormalities. When cells are irradiated with a laser or light-emitting diode (LED) of approximately 405 nm, PpIX exhibits red fluorescence at 635 nm. Thus, a cancer lesion can be identified using this property of PpIX. Accordingly, PDD can be applied for tumor detection and might be useful in identifying EGC, regardless of the endoscopist’s level of experience; however, its usefulness with flexible endoscopy remains unestablished owing to the limited number of studies (9).

The endoscopic miss rate of EGC detection is often higher than desired. According to Menon et al., 11.3% of upper gastrointestinal cancers are missed during endoscopy examination for up to 3 years before diagnosis (10). Shimodate et al. re-examined previous endoscopic reports of patients with EGC and found that 75.2% of EGCs had not been recorded in previous esophagogastroduodenoscopy reports but were evident upon the review of endoscopic photographs (11). In the current retrospective study, we investigated the diagnostic yield of PDD for identifying multiple gastric cancers and high-grade adenomas.

## Materials and Methods

### Patients

In total, 44 patients underwent 5-ALA-mediated PDD for 53 target lesions of endoscopic resection at university hospitals from December 2013 to March 2019. The inclusion criteria were as follows: (1) patients aged 20 years or older who agreed to participate in the study, (2) patients who were to undergo endoscopy, and (3) patients for whom the Eastern Cooperative Oncology Group Performance Status (PS) was 0 or 1. Patients were excluded if they had an allergy to 5-ALA or porphyria and/or were taking medication known to cause photosensitivity or ingesting foods containing St. John’s wort. Patients with any other concurrent tumor, those who were pregnant, and/or those who had severe dysfunction due to heart, liver, lung, or renal disease were also excluded. Finally, patients who had a doctor-in-charge who believed they were not appropriate candidates for the study were excluded. The study was approved by the institutional ethics committees (#11032827 and #17B013). Written informed consent was obtained prior to study enrolment from patients who met the inclusion criteria but did not meet the exclusion criteria. One patient, as described in the Results section, was excluded after enrollment, leaving a final study cohort of 43 patients and 52 lesions.

### 5-ALA PDD

Endoscopic PDD was performed 3 h after the oral administration of 20 mg/kg 5-ALA. For the *in vivo* fluorescence detection of PpIX accumulation, a Sie-P1video image endoscope system (Fuji Film Medical Co., Tokyo, Japan) consisting of a VP-0001 processor, an LL-4450-P1 light source, and an XG-0002-P1 scope or a Sie-P2 system (Fuji Film Medical Co.) with a VP-7000-P2 processor, an LL-7000-P2 light source, and an EG-L590ZW esophagogastroduodenoscopy scope was used (12, 13). A 410-nm laser was used for blue-light excitation of PpIX to induce red fluorescence emittance. When a fluorescence signal was visualized and confined to the tumor and not in the surrounding non-tumorous tissue, it was referred to as PDD-positive. After identifying the lesion, the stomach cavity was evaluated followed by endoscopic submucosal dissection (ESD) of the target lesion(s). Thus, endoscopic PDD was performed on the day of ESD immediately prior to performing the ESD procedure. Whereas ESD was performed for lesions with a nominal risk of lymph node metastasis, in accordance with previously described criteria (14), five patients underwent standard surgery with the removal of regional lymph nodes. All patients were shielded from strong light, such as direct sunlight, for 24 h following the 5-ALA PDD procedure to avoid potential phototoxic reactions.

### Histopathological Evaluation

The resected lesions were classified according to the English edition of the Japanese Classification of Gastric Carcinoma (15). The tumor characteristics described included the tumor location, macroscopic type, size, and histology, as well as the depth of tumor invasion. Tumor location was categorized as the upper, middle, or lower thirds of the stomach. Macroscopic tumors are categorized as elevated or flat/depressed types. Tumor histology was classified as adenoma, differentiated adenocarcinoma [well-differentiated adenocarcinoma (tub1), moderately differentiated adenocarcinoma (tub2), or papillary adenocarcinoma pap)], or undifferentiated adenocarcinoma [poorly differentiated adenocarcinoma (por) or signet ring cell carcinoma (sig)]. Biopsy sampling and histopathological evaluation were performed once new PDD-positive lesions were found.

### Statistical analysis

The statistical significance of comparisons between groups was determined using the *χ* ^2^ test and Student’s *t*-test. Statistical significance was set at *p* < 0.05.

## Results

### Clinicopathological Characteristics

After enrolment, one patient who had received anticancer drug treatment until 1 month prior to completing the consent form was excluded from the study in accordance with the exclusion criterion. In total, 52 lesions from the remaining 43 patients were included in this study. The characteristics of the 43 patients and 52 lesions are shown in **Table 1**. The median age of the patients was 72 years (range, 56–77 years), there were 32 men, and all patients had a PS of 0. The 52 lesions analyzed included 42 adenocarcinomas and 10 high-grade adenomas. There were 26 elevated lesions (24 macroscopic type 0–IIa lesions; two macroscopic type 0–I lesions) and 26 flat/depressed lesions (23 macroscopic type 0–Ic lesions; three macroscopic type IIb lesions). Of the 42 cancer lesions, 37 were intramucosal adenocarcinomas (T1a) and five had infiltrated into the submucosa (T1b). Of the 42 adenocarcinomas, four were signet ring cell carcinomas. In **Table 1**, 52 lesions consisted of 42 gastric cancer lesions and 10 adenomas. There was neither lymphatic nor venous invasion in the 10 adenomas; 42 cancerous lesions were assessed as for lymphatic and venous invasion. There were three lesions with lymphatic invasion, each being ly1, and three lesions with venous invasion, each being v1. One case had both the ly1 and v1. The median tumor size of the excised lesions was 18.5 mm in diameter with an interquartile range (IQR) of 10.0–30.0. In the case series, all ESD and surgery with removal of the lymph nodes were successfully conducted uneventfully. Histopathological assessment did not identify any lymph node metastasis.

**Table 1:** Characteristics of 52 lesions in 43 patients with early gastric cancer and/or adenoma who underwent endoscopic photodynamic diagnosis (PDD).

Baseline characteristics	Total samples (*n* = 52)	
Age, years (median, interquartile range)	72.0	(64.0–77.0)
Sex, male/female	32/11	
Endoscopy, Sie-P1/Sie-P2	30/13	
Tumor size, mm (median, interquartile range)	18.5	(10.0–30.0)
Tumor location in the stomach, *n* (%)		
Upper third	6	(11.5)
Middle third	29	(55.8)
Lower third	17	(32.7)
Macroscopic type, *n* (%)		
0–I	2	(3.8)
0–IIa	24	(46.2)
0–IIb	3	(5.8)
0–IIc	23	(44.2)
Histological classification, *n* (%)		
Adenoma	10	(19.2)
Tub	38	(73.1)
Sig	4	(7.7)
Depth of tumor invasion, *n* (%)		
T1a	37	(71.2)
T1b	5	(9.6)
Adenoma	10	(19.2)
Lymphatic invasion, *n* (%)		
ly0	39	(92.9)
ly1	3	(7.1)
Venous invasion, *n* (%)		
v0	39	(92.9)
v1	3	(7.1)
5-Aminolevulinic acid-mediated PDD, *n* (%)		
Negative	7	(13.5)
Weakly positive	24	(46.2)
Strongly positive	21	(40.3)

### Detection Rate and Intensity Levels of 5-ALA PDD Fluorescence

We detected 5-ALA-mediated PpIX fluorescence in 45 of the 52 lesions that were initially intended for PDD, resulting in a detection rate of 86.5%; the seven lesions, including four signet ring cell carcinomas, not identified by 5-ALA PDD should be deemed misdiagnoses. On the other hand, we took biopsies from six areas that we suspected as new lesions. While 4 lesions were gastric neoplasms resected endoscopically, another two lesions were normal.

**Figure 1:**
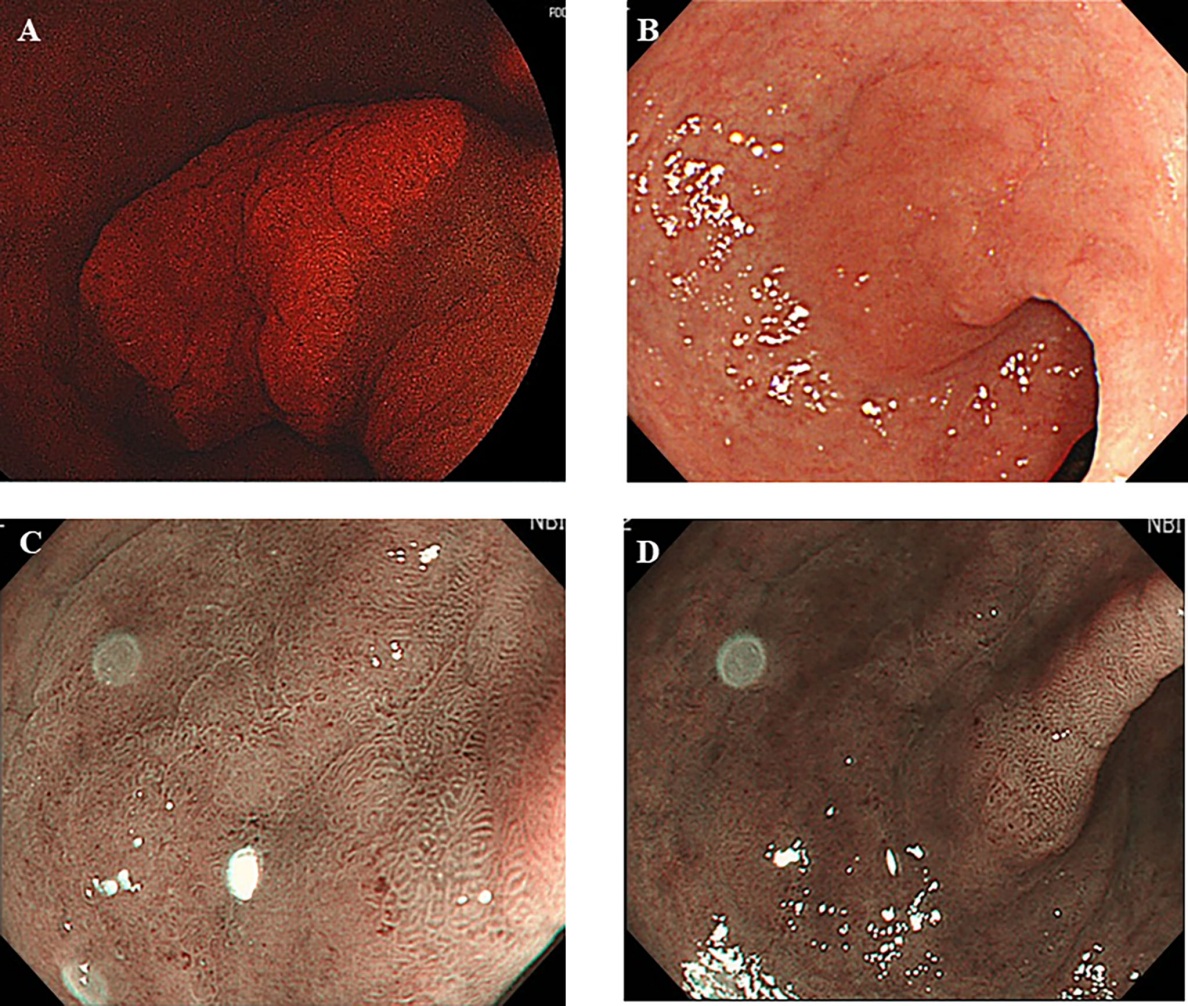
Five aminolebulinic acid-mediated photodynamic diagnosis (5-ALA PDD)-positive lesion **(A)** that were not clearly identified by conventional white light imaging **(B)** only with slightly elevation and narrow band imaging with **(C)** or without **(D)** magnification without apparent demarcation during previous examination.

Forty-two lesions were previously assessed by magnifying IEE with narrow band imaging (NBI) before 5-ALA PDD, and 38 lesions (90.5%) were identified by the magnifying IEE with NBI. There were four PDD-negative cases among the 38 lesions. On the other hand, there were 3 PDD-positive lesions that were not identified by combination of conventional endoscopy and IEE during previous examinations (**Figure 1**). Each was determined to be differentiated intramucosal adenocarcinoma, which could be subsequently resected using ESD.

We summarize the sensitivity and the false-positive rate for 5-ALA PDD and the sensitivity for magnifying IEE with NBI when the results of pathological validation were considered as gold standard (**Table 2**).

**Table 2:** Summary of the sensitivity and the false-positive rate for the 5-aminolevulinic acid-mediated photodynamic diagnosis (PDD) and the sensitivity for the magnifying image-enhanced endoscopy when the results of pathological validation were considered as gold standard.

	Sensitivity	False-positive rate
**5-aminole vulinic acid-mediated PDD**	66.7% (4/6)* 86.5% (45/52)	33.3% (2/6)*
**Magnifying image-enhanced endoscope**	90.5% (38/42)	N/A

*new lesions, N/A, not applied.

**Figure 2:**
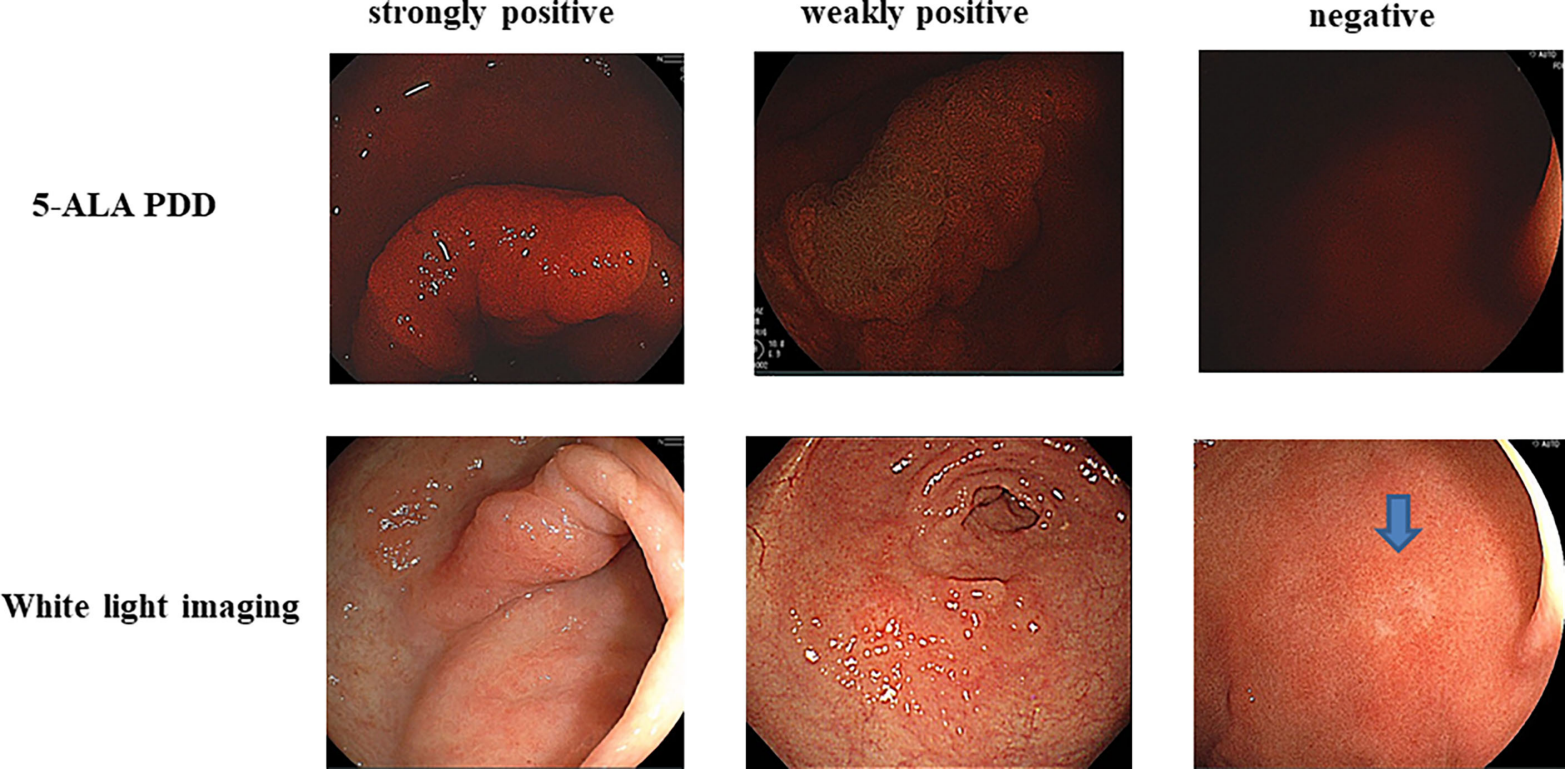
Strongly positive (++) 5-aminolevulinic acid-mediated photodynamic diagnosis (5-ALA PDD) and weakly PDD-positive (+) and PDD-negative images are shown. The each corresponding white light imaging was shown in lower parts.

As there was a difference in the level of fluorescence among the 45 lesions, the PDD-positive lesions were divided into strongly PDD-positive (++) and weakly PDD-positive (+). Tumors were deemed PDD-positive (++) when the intense fluorescence and diffuse fluorescence were clearly greater compared to the background signal and that of the PDD-positive (+) samples (12, 13). There were 21 PDD-positive (++) lesions and 24 PDD-positive (+) lesions (**Table 1**). Representative images are shown in **Figure 2**. Thirty-eight intestinal-type gastric cancers included three PDD-negative, 19 PDD-positive (+), and 16 strongly positive lesions. Ten high-grade adenomas included five PDD-positive (+) and five PDD-positive (++) lesions. There were no significant differences in the fluorescence intensity between intestinal-type gastric cancers and high-grade adenomas. Again, 37 T1a intramucosal cancers included six PDD-negative, 17 weakly positive, and 14 PDD-positive (++) lesions, whereas 5 T1b gastric cancers included one PDD-negative, two PDD-positive (+), and two PDD-positive (++) lesions. Five tumors had lymphatic or vascular involvement, and there were one PDD-negative, two PDD-positive (+), and two PDD-positive (++) lesions.

### PDD for Multiple Lesions

In eight patients with multiple lesions, 17 lesions were identified *via* 5-ALA-mediated PDD. The patient and lesion characteristics for these eight patients are shown in **Table 3**. The median age of the patients was 73 years (range 64–75 years), and there were seven men. There were nine differentiated intramucosal adenocarcinomas (T1a) and eight high-grade adenomas. There were no undifferentiated adenocarcinomas, including any signet ring cell carcinomas, despite the statistical insignificance. There were 12 elevated lesions (all macroscopic type 0–IIa) and five flat/depressed lesions (four lesions were macroscopic type 0–IIc; one lesion was macroscopic type IIb macroscopic type). In **Table 3**, 17 lesions consisted of 9 gastric cancer lesions and 8 high-grade adenomas. There was neither lymphatic nor venous invasion in the 8 adenomas; 9 cancerous lesions were assessed as for lymphatic and venous invasion, and all were negative. All 17 lesions were curatively resected using ESD, with nominal lymphovascular invasion and without residual lesions.

**Table 3:** Characteristics of 17 multiple lesions in eight patients with early gastric cancer and/or adenoma.

Baseline characteristics	Total samples (*n* = 17)	
Age, years (median, interquartile range)	72.5	(64.0–75.5)
Sex, male/female	7/1	
Tumor size, mm (median, interquartile range)	23.0	(10.0–30.0)
Tumor location in the stomach, *n* (%)		
Upper third	2	(11.8)
Middle third	10	(58.8)
Lower third	5	(29.4)
Macroscopic type, *n* (%)		
0–IIa	12	(70.6)
0–IIb	1	(5.9)
0–IIc	4	(23.5)
Histological classification, *n* (%)		
Adenoma	8	(47.1)
Tub	9	(52.9)
Depth of tumor invasion, *n* (%)		
T1a	9	(52.9)
Adenoma	8	(47.1)
Lymphatic invasion, *n* (%)		
ly0	9	(100.0)
ly1	0	(0)
Venous invasion, *n* (%)		
v0	9	(100.0)
v1	0	(0)
5-Aminolevulinic acid-mediated PDD, *n* (%)		
Negative	0	(0)
Weakly positive	5	(29.4)
Strongly positive	12	(70.6)

### Adverse Events in Relation to PDD

There was a transient elevation of circulating aspartate aminotransferase to levels > 100 IU/L in seven patients, including five grade 1 and two grade 2 events according to Common Terminology Criteria for Adverse Events (CTCAE) grades (http://www.jcog.jp/doctor/tool/CTCAEv5J_20180730_v21_0.pdf). Grade 1 hypotension was observed in two patients, both of whom recovered spontaneously. Photosensitivity was not observed in any of the patients.

## Discussion

The present study demonstrated that laser-based 5-ALA PDD could be used to successfully detect gastric cancers and adenomas that can simultaneously originate in the gastric cavity, mainly in the middle and lower compartments (**Table 1**). Nearly 20% of EGC cases (8/43) had multiple adenocarcinoma and/or adenoma lesions. Gastric adenoma is essentially a benign lesion, whereas intestinal-type gastric cancers have a possible risk of metastasis even in the earlier stages. As each of the 10 lesions were diagnosed as high-grade adenoma, we performed ESD for the purpose of total resection considering the known risks of malignant transformation and possibly having malignant foci in the precancerous lesion (16). Among 52 lesions that were assessed by 5-ALA PDD, 45 lesions were judged positive; the detection rate could be 86.5%, but it might be attributable to the inclusion criteria of ESD; cases of precancerous high-grade adenomas or differentiated adenocarcinoma with nominal risks of lymph node metastasis were preferentially enrolled. Future studies are necessary in order to evaluate whether the use of 5-ALA PDD in combination with ESD would be useful on the basis of a positive PpIX fluorescence. On the other hand, we took biopsies from six areas that we suspected as new lesions. While 4 lesions were gastric neoplasms resected by ESD, another two lesions were normal. The sensitivity of PDD could be 66.7% (4/6) when it is difficult to determine whether a tissue is a lesion in bright-field view. In other words, false positivity might reach 33.3% (2/6). When examiners can detect the fluorescence rather objectively, the rates of false positives would be almost comparable between experts and inexperienced endoscopists if biopsy sampling can be conducted appropriately.

The underlying mechanism associated with the differential accumulation of 5-ALA-induced photosensitizing PpIX in tumor cells, including gastric cancer cells, remains uncertain. Nevertheless, the histopathological types of gastric cancer (intestinal vs. diffuse; differentiated vs. undifferentiated) could affect the red fluorescence intensity of PpIX in endoscopic PDD upon excitation by light with a wavelength of 410 nm. Thus, tumor cells could be detected using fluorescence navigation. As expected, most differentiated-type gastric cancers emitted diverse degrees of red fluorescence, peaking at 635 nm, whereas pure signet ring cell carcinoma under the same conditions emitted nominal fluorescence in each case (17). These results suggest substantial differences among diverse gastric tumors in the accumulation of PpIX, a metabolite produced by the heme biosynthetic pathway, following 5-ALA administration (18). The production of PpIX through the porphyrin biosynthesis pathway is promoted in tumor cells because of the activation of the porphyrin synthetic enzyme following the incorporation of 5-ALA, which occurs *via* the peptide transporter oligopeptide transporter-1 (19). As a result, PpIX accumulates in tumor cells, including intestinal-type gastric tumors. Immunohistochemical assessment has revealed that the two porphyrin synthetic enzymes, coproporphyrinogen-III oxidase and protoporphyrinogen oxidase, are highly expressed in the cytoplasm of differentiated gastric cancer cells, whereas both enzymes are downregulated in signet ring cell carcinoma cells (17, 20). Similarly, the expression of peptide transporter oligopeptide transporter-1 is higher in the membranous surface of differentiated gastric cancer cells than in signet ring cell carcinoma cells (17, 19). According to immunohistochemical studies, this transporter protein is highly expressed in differentiated-type tumors, leading to strong red fluorescence upon photodynamic diagnosis, which might be related to the absorbance and accumulation of photosensitizing PpIX in typically differentiated intestinal-type gastric epithelial neoplasms. The ATP-binding cassette transporter ATP-binding cassette subfamily G member 2 can also affect intracellular photosensitizing PpIX levels in gastric cancer cells *in vitro*. Although the expression of ATP-binding cassette transporter ATP-binding cassette sub-family G member 2 can be observed in both types of lesions, a loss of polarity was previously observed in undifferentiated tumors (17). Additional studies are needed to determine the meaning of this result.

IEE, including NBI and blue-light imaging, highlights the mucosal surface structure and vascularity when using magnifying endoscopy. Thus, magnified endoscopy with IEE allows for the identification of subtle superficial abnormalities in the early stages of gastric cancer that are difficult to recognize using conventional white-light endoscopy (21–25). Currently, NBI-magnifying endoscopy is widely used for the differential diagnosis of gastric cancer and gastritis and to accurately identify cancer margins. Owing to the development of magnifying endoscopy with IEE, the diagnostic accuracy for gastric neoplasm lesions has improved by implementing the presence/absence of a demarcation line and irregular micro-surface patterns and irregular microvascular patterns. Studies on the diagnostic performance of magnifying IEE using the vessel plus surface (VS) classification have shown accurate diagnostic yields for EGC, and the standardized algorithm named the Magnifying Endoscopy Simple Diagnostic Algorithm for Early Gastric Cancer (MESDA-G) was established (24, 26, 27). In the current cohorts, 42 lesions were previously assessed by magnifying IEE with NBI before 5-ALA PDD, and 38 lesions (90.5%) were identified by magnifying IEE with NBI. There were four cases that were negative for 5-ALA PDD, confirming the standard method of detecting gastric neoplasm lesions by utilizing magnifying IEE. However, inflammation associated with *H. pylori* infection makes qualitative diagnosis difficult and subjective, even among expert endoscopists (26, 28). According to a Japanese multicenter study, NBI does not contribute to an increase in the detection rate of EGC and does not outperform standard white light imaging (29). Indeed, eradication therapy against *H. pylori* infection has been widely performed; however, the incidence of gastric cancer has not decreased. Moreover, gastric cancer post-eradication of *H. pylori* infection often resembles gastritis, making the diagnosis of EGC difficult (27, 30–32). Therefore, it is desirable to develop an endoscopic diagnostic method that enables an objective evaluation, irrespective of the expertise of the endoscopists. Our current results suggest that 5-ALA PDD might be useful in identifying typical intestinal types of gastric cancers in the early stages of disease or even as pre-cancerous lesions. However, undifferentiated signet ring cell carcinomas seem unsuitable for identification *via* this molecular imaging methodology. However, the number of diffuse-type gastric cancer cases in our current study was too small to demonstrate clinical significance. Forty-two lesions were initially assessed by magnifying IEE with NBI before ESD and 5-ALA PDD.

The use of 5-ALA PDD has proven useful for glioblastoma removal (33), and 5-ALA was approved as an intraoperative diagnostic agent for malignant glioma surgery by the US Food and Drug Association in 2017 (34). For bladder cancer, ALA-PDD is superior to conventional endoscopic diagnoses performed under white light for the detection of flat lesions, even within carcinoma *in situ* (7, 35–38). This agent was also approved in 2017 for the intraoperative visualization of non-muscle-invasive bladder cancer during the transurethral resection of bladder tumors. The detection rate of additional tumors using 5-ALA PDD ranges from 10% to 30%, ultimately improving the survival rates of patients (39–44). In the surgical field for gastric cancer, 5-ALA PDD has been reported to improve the detection rate of peritoneal dissemination by 10% compared to that with white light (45). We also found new lesions in our current study when 5-ALA PDD was applied, which indicates that the potential for under-detecting EGC or precancerous adenoma lesions could be reduced by using PDD. Thus, it would be expected that 5-ALA PDD exerts additional effects over white light imaging. Additional well-designed prospective studies are warranted to assess whether 5-ALA PDD outperforms white light imaging for the accurate and additional detection of gastric neoplastic lesions. Again, additional gastric epithelial neoplasm lesions that were previously overlooked with initial conventional endoscopy and IEE were identified *via* 5-ALA PDD. Whereas IEE is widely used for gastric cancer detection, the full magnifying function that is necessary to obtain an even closer and clearer visualization of microvascular and microsurface patterns might not be routinely available for each lesion and in the vast gastric cavity.

We believe that 5-ALA-PDD could help search for gastric epithelial neoplasms that can be resected endoscopically, EGC, and high-grade adenoma in the initial IEE, providing additional diagnostic yields to detect such multiple lesions simultaneously, but further research is necessary to address whether 5-ALA PDD can help identify EGC in the initial IEE. We considered maintaining a degree of insufflation throughout the examination so that all anatomical areas can be properly distinguished throughout the vast gastric cavity. A combination of close-up and panoramic views is essential to achieve a complete examination and to register the total mucosal lining, but we encountered reduction of the fluorescence during the blue light irradiation after exceeding 5 min, which might be referred to as photo-bleaching (12); the durable close-up photo-documentation should be avoided. Again, the amount of excitation light per unit area irradiated to the lesion is inversely proportional to the square of the distance from the light source to the lesion, meaning that the intensity of PpIX fluorescence observed in the lesion might depend on the distance between the light source and the lesion; however, it is beyond the scope of the current study.

**Figure 3:**
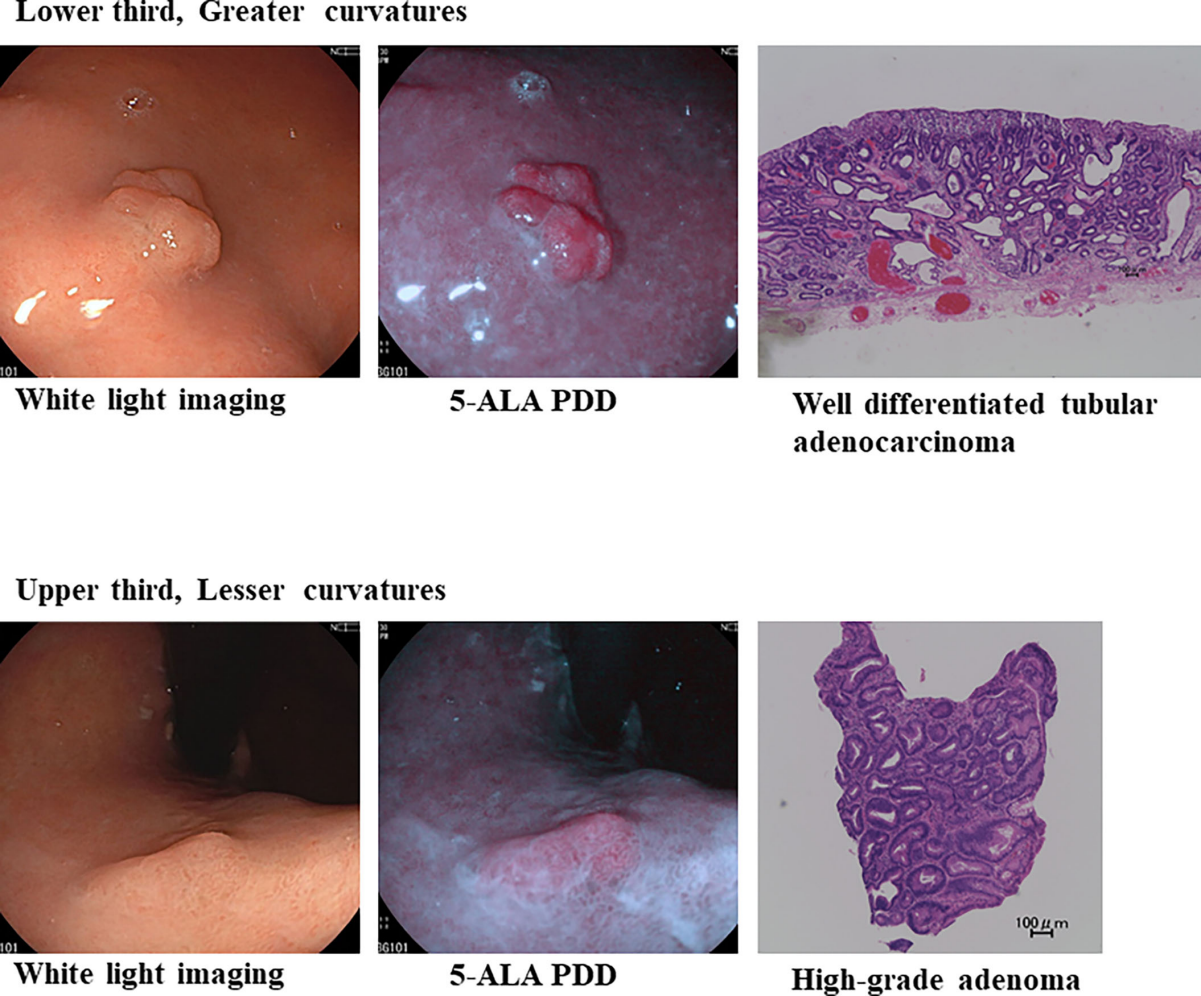
5-ALA PDD using an endoscope with a light-emitting diode can identify well-differentiated adenocarcinoma-positive (++) and high-grade adenoma-positive (+) lesions in the same patient.

This study had several limitations. It was a retrospective study with a relatively small cohort and was conducted at a limited number of tertiary university hospitals. Non-experts were not enrolled in the current study, and the examiners were experts, who were exceptional at safely conducting ESD and who have handled at least 50 cases of ESD. Therefore, future studies are warranted to explore diverse clinical settings to assess to what extent the combined use of 5-ALA PDD can increase the detection rate of lesions that inexperienced endoscopists may miss during bright-field imaging. In addition, *H. pylori* infection status was not systemically confirmed. PDD-negative signet ring cell carcinomas were exclusively negative for *H. pylori* infection. Considering that most gastric cancers, particularly intestinal-type gastric cancers, are associatied with a predisposition as a result of a current or past *H. pylori* infection (46, 47), 5-ALA PDD could be used to identify EGC of differentiated gastric adenocarcinoma related to *H. pylori* infection. This suggests future directions for clinical trials to confirm the diagnostic yield of 5-ALA PDD, targeting such lesions. Given the high incidence and mortality rates of gastric cancer, particularly in Asia, it is of clinical significance to detect gastric cancer in the early stages with nominal risks of lymph node metastasis, at which point most cases can be cured using minimally invasive endoscopic resection. This is supported by the fact that almost all lesions identified by 5-ALA PDD in our case series were extensively resected using ESD with no residual cancer. Furthermore, as shown in the current study, detectable fluorescence was not emitted by any of the signet ring cell carcinomas. This might have been due to the excitation light used for the PDD. Owing to the short wavelength of the LED light, it could have been attenuated in the superficial layer of the mucosa, thereby not reaching the deeper mucosal layer where signet ring cell carcinomas arise (17, 20). When the LED light was equipped into a Fujifilm ELUXEO Endoscopic Imaging System that is readily launched, a stronger light intensity is safely produced for 5-ALA PDD. In fact, we successfully identified multiple lesions of differentiated EGC and adenoma using this system (**Figure 3**). Whether the defined region in the PDD image was PDD-positive or PDD-negative was visually determined by examiners. Artificial intelligence assistance would provide a more objective and ultimately a more reliable identification of lesions, thereby reducing the potential of overlooking lesions, even when using 5-ALA PDD. Of note, seven patients had elevated circulating aspartate aminotransferase after 5-ALA-PDD, and thus, this method might not be used for patients with liver diseases. Finally, there was no significant difference in 5-ALA PDD fluorescence intensity between T1a and T1b and between lymphatic or vascular involvement. The aim of this study was to assess diagnostic yields of 5-ALA PDD to detect earlier stages of EGC and precancer lesions, and there were nominal cases of advanced gastric cancers enrolled in the study cohorts. Further studies are needed to address the utility of 5-ALA PDD in association with more aggressive biological behavior.

**Figure 4:**
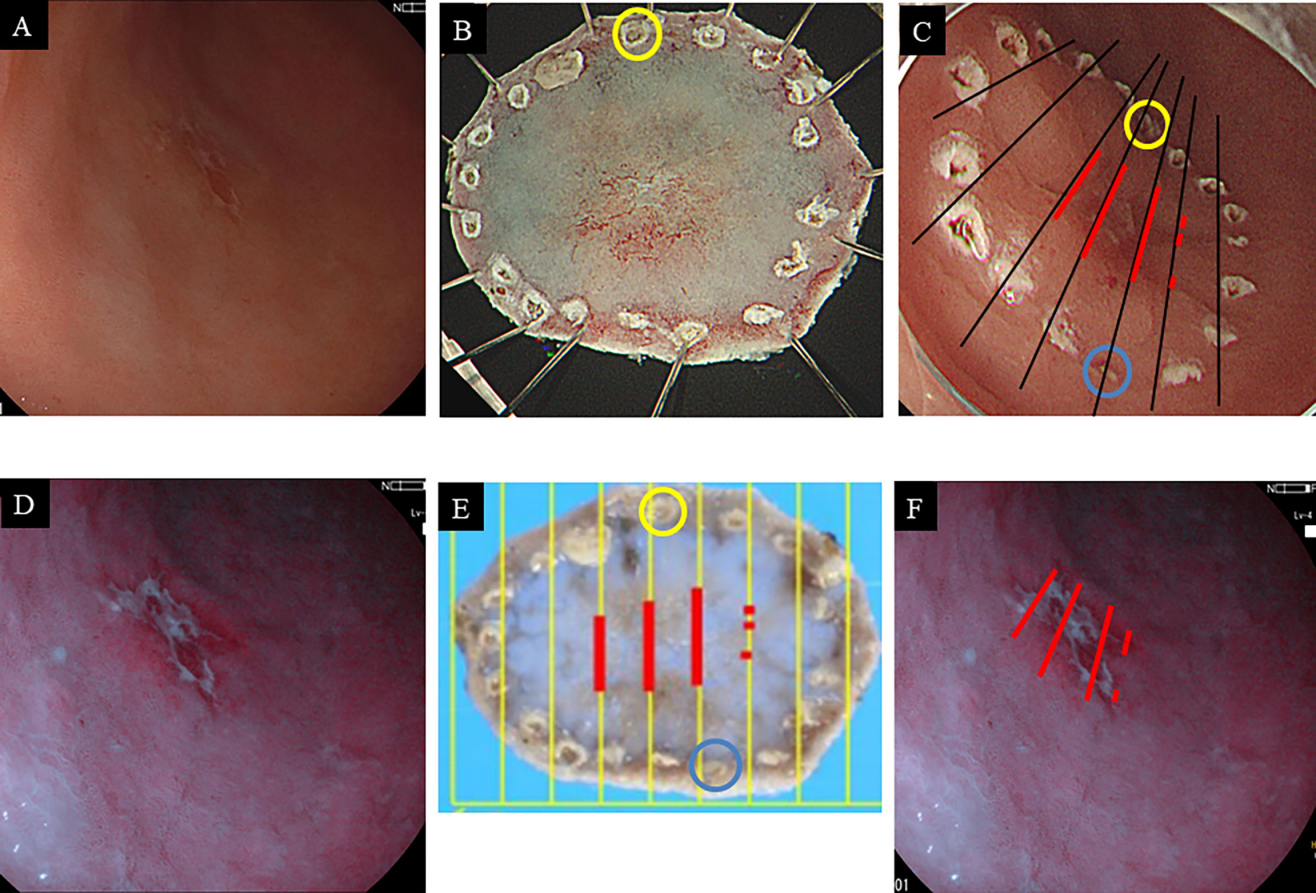
Endoscopic images of gastric cancer on the anterior wall of the antrum with white light imaging **(A)** and 5-ALA PDD **(B)**. Macroscopic findings of the resected specimen by endoscopic submucosal dissection before formalin fixation **(C)** and after formalin fixation **(D)**. Each blue and yellow circle in panels **(C, D)** corresponds to a respective circle in the endoscope image **(E)**. The red lines correspond to the extent of cancer cells. The cancerous horizontal extent in histopathology may be coincident with the PDD-positive area **(F)**.

In conclusion, we confirmed that 5-ALA PDD could be used to identify intestinal types of gastric neoplasms, EGC, and high-grade adenoma. This approach, based on our current protocol, should provide additional diagnostic yields to safely detect multiple lesions simultaneously. Accordingly, 5-ALA PDD will likely become a useful diagnostic method for gastric cancer following the execution of larger well-designed multicenter studies. Again, 5-ALA PDD may contribute to increasing the rate of negative resection margins (**Figure 4**). Although there are many hurdles to overcome for the clinical application of 5-ALA PDD in ESD, this study is commendable in that it shows the potential of 5-ALA PDD as a diagnostic test for intestinal-type gastric cancer.

## Data Availability Statement

The raw data supporting the conclusions of this article will be made available by the authors, without undue reservation.

## Ethics Statement

The studies involving human participants were reviewed and approved by Tottori University and Nagasaki University Hospital. The patients/participants provided their written informed consent to participate in this study.

## Author Contributions

Writing of the original manuscript draft, HK and TS. Writing—review and editing of the manuscript, YI and AY. Collecting data, KH, TY, and MF. Analysis, TY, HK, and TS. Supervision of the study, HI. All authors have contributed to the manuscript and approved the submitted version.

## Conflict of Interest

The authors declare that the research was conducted in the absence of any commercial or financial relationships that could be construed as a potential conflict of interest.

## Publisher’s Note

All claims expressed in this article are solely those of the authors and do not necessarily represent those of their affiliated organizations, or those of the publisher, the editors and the reviewers. Any product that may be evaluated in this article, or claim that may be made by its manufacturer, is not guaranteed or endorsed by the publisher.
